# Hepatitis E Virus and Porcine-derived Heparin

**DOI:** 10.3201/eid1904.121792

**Published:** 2013-04

**Authors:** C. Crossan, L. Scobie, J. Godwin, J.G. Hunter, T. Hawkes, H.R. Dalton

**Affiliations:** Glasgow Caledonian University, Glasgow, Scotland, UK (C. Crossan, L. Scobie, J. Godwin);; Royal Cornwall Hospital, Truro, UK (J.G. Hunter, T. Hawkes, H.R. Dalton);; University of Exeter Medical School, Truro (H.R. Dalton)

**Keywords:** hepatitis E, HEV, heparin, porcine, porcine parvovirus, porcine circovirus 2, pharmacovigilance, viruses, zoonoses

**To the Editor:** Cases of sporadic, locally acquired hepatitis E have been increasingly identified in industrialized countries over the last few years (*1*). In this setting, hepatitis E is thought to be a zoonotic infection, with pigs as the primary host. Consumption of uncooked or lightly cooked pork meat products is thought to be a key route of infection, but other routes of transmission have been documented (*2*). For example, there have been several iatrogenic cases after transfusion of hepatitis E virus (HEV)–contaminated blood products (*3*) and transplantation of an HEV-infected donor liver (*4*). However, in most cases the source and route of infection are uncertain.

In May 2011, a 42-year-old woman sought care at the Royal Cornwall Hospital in Truro, United Kingdom, for a 1-week history of malaise, diarrhea, nausea, and vomiting. Physical examination results were normal. Her liver function test results, however, indicated hepatitis: alanine aminotransferase 2,785 IU/L (reference range 10–36 IU/L), alkaline phosphatase 319 IU/L (reference range 30–130 IU/L), and bilirubin 30 μmol/L (reference range <21 μmol/L). HEV IgM and IgG serologic test results for the patient were positive, and HEV genotype 3 was identified in her blood by reverse transcription PCR and sequencing. Other causes of viral hepatitis and hepatocellular jaundice, including hepatitis viruses A, B, and C; Epstein-Barr virus; and autoimmune hepatitis, were excluded by testing. As with most immunocompetent persons with HEV, the patient made an uneventful clinical recovery after 12 weeks, and her liver function tests returned to normal after 8 weeks.

The source and route of infection in this case was uncertain. A detailed in-person assessment of potential risk factors was undertaken with the patient. She had not traveled outside the United Kingdom in the previous 3 months. She rarely ate pork products (well cooked bacon only); ate no shellfish; and had no workplace, domestic, or recreational exposure to pigs or their effluent. However, 4 weeks before symptom onset, the patient had acute appendicitis for which she underwent an uneventful laparoscopic appendectomy and was hospitalized for 2 days. During hospitalization she received no blood products, but, as prophylaxis for thromboembolic disease, she received 2 doses (5,000 IU each) of low–molecular weight heparin (Fragmin [dalteparin sodium]; Pfizer, Sandwich, UK) by subcutaneous injection. All heparins used in Europe and North America are isolated from porcine intestinal mucosa (*5*). The exact purification methods used by heparin manufacturers are deemed commercially sensitive and not in the public domain, so it is impossible to evaluate whether the isolation process would be sufficient to remove or inactivate any contaminating HEV. The virus is known to be acid and alkaline stable; heat sensitivity varies, depending on strain and heating conditions, although heating at 60°C for 1 hour is generally sufficient to achieve 96% inactivation (*6*). To our knowledge, no investigation has determined whether clinical-grade heparin could contain viral contaminants. Thus, we hypothesized that the heparin the patient received might have been the source of her HEV infection.

To examine this possibility, we screened multiple batches of hospital pharmacy–grade heparin for the presence of HEV, including batches of dalteparin sodium that were in use at the hospital when the patient received treatment for appendicitis. Before testing, the samples were ultracentrifuged to concentrate any contaminating virus and enable the removal of excipients, which could inhibit the assay. We tested samples by quantitative reverse transcription PCR (*7*) in parallel with positive World Health Organization HEV RNA standard spiked controls, which showed the limit of detection (LOD) to be 500 IU/mL, regardless of the heparin’s excipient or concentration. This LOD is within the range used by collaborating laboratories in the establishment of the World Health Organization HEV RNA standard (http://whqlibdoc.who.int/hq/2011/WHO_BS_2011.2175_eng.pdf). In addition, we tested the heparin samples for porcine circovirus 2 (PCV2), an identified adventitious agent of several rotavirus vaccines (*8*) and porcine parvovirus (PPV) (*9*), a known contaminate of porcine clotting factor hyate:C (*10*). Although samples were tested in parallel with PCV2- and PPV- positive spiked controls, we were unable to calculate the LOD for these assays because international standards are not available for these viruses.

All samples tested negative for HEV, PCV2, and PPV (Table), which would indicate the patient’s source of HEV infection is unlikely to have been the heparin. However, we cannot rule out low-level viral contamination below the sensitivity of the assay. We also cannot exclude that the negative test results were related to the Poisson effect. Given that all samples analyzed were negative for all 3 viruses tested, it seems likely that the heparin manufacturing process is sufficient to remove viral contaminants. However, this may not necessarily be the case for other porcine-derived products, such as porcine insulin, factor VIII C, pancreatin, and poractant alfa. Further investigation is warranted to exclude these products as potential sources of HEV infection.

**Table:** Heparin samples tested for hepatitis E virus, porcine circovirus 2, and porcine parvovirus*

Producer, proprietary name/other names, batch or lot no.	Use	Excipient	Concentration	Quantity tested, IU	95% upper CL, IU^−1^†
Sanofi‡					
Clexhane/enoxaparin	Injection	H_2_O			
ILA01			20 mg/0.2 mL	6,000	0.0006
34751			40 mg/0.4 mL	4,000	0.0009
OLC56			80 mg/0.8 mL	8,000	0.0005
ILA53			60 mg/0.6 mL	6,000	0.0006
OLC07			100 mg/ mL	10,000	0.0004
12255			120 mg/0.8 mL	12,000	0.0003
Pfizer§					
Fragmin/dalteparin sodium	Injection	H2O pH adjusted with HCl or NaOH			
12339A01			5,000 IU/0.2 mL	15,000	0.0002
12338A01			5,000 IU/0.2 mL	15,000	0.0002
12327B01			5,000 IU/0.2 mL	15,000	0.0002
12257A01			5,000 IU/0.2 mL	15,000	0.0002
12444A01			5,000 IU/0.2 mL	10,000	0.0004
12122C01			7,500 IU/0.3 mL	7,500	0.0005
74774D51			10,000 IU/0.4 mL	10,000	0.0004
74871B51			12,500 IU/0.5 mL	25,000	0.0001
74779G51			12,500 IU/0.5 mL	12,500	0.0003
74871B51			12,500 IU/0.5 mL	12,500	0.0003
74743C52			15,000 IU/0.6 mL	30,000	0.0001
74755A51			15,000 IU/0.6 mL	30,000	0.0001
74832A52			15,000 IU/0.6 mL	15,000	0.0002
74832A01			15,000 IU/0.6 mL	15,000	0.0002
X08580	¶	¶	100,000 IU/4 mL	100,000	0.00004
Wockhart#					
Monoparin	Injection	H2O pH adjusted with HCl or NaOH			
PK40319			1,000 IU/mL	20,000	0.0002
3090			1,000 IU/mL	10,000	0.0004
Hepsal	Flushing	NaCl, H2O, HCl, and NaOH			
5000090			10 IU/mL	120	0.03
91180			50 IU/mL	50	0.07
1069			200 IU/mL	200	0.02
Leo**					
Heparin sodium	Intravenous flushing	Benzyl alcohol, methyl parahydroxybenzoate, propyl parahydroxybenzoate, sodium citrate, NaCl, and H_2_O			
DD7314			100 IU/mL	200	0.02
CC4338			100 IU/mL	200	0.02
Celgene††,					
Refludan/Lepirudin, 25561611A‡‡	Powder used for solution for injection/infusion	Mannitol, NaOH, and H_2_O	12.5 mg/mL	NA	NA
Total quantity tested	NA	NA	NA	404,270	0.000009

*NA, not applicable. †The 95% upper confidence limit of the probability of a virus-positive result per IU was calculated on the basis of the quantity tested for each batch. This was estimated, assuming perfect detection of a Poisson process, by using Fisher exact test. For the pooled result, the upper 95% estimate is ≈1 per 100,000 IU. ‡Sanofi (Guilford, UK). §Pfizer (Sandwich, UK). ¶Multidose vials used for injection, Excipients: Benzyl alcohol and H_2_O. #Wockhart (Wrexham, UK). **Leo (Buckinghamshire, UK). ††Celgene (Uxbridge, UK). ‡‡Non-porcine–derived anticoagulant alternative.
